# Breast milk delivery of an engineered dimeric IgA protects neonates against rotavirus

**DOI:** 10.1016/j.mucimm.2025.01.002

**Published:** 2025-04

**Authors:** Stephanie N. Langel, Claire E. Otero, Justin T. Steppe, Caitlin A. Williams, Tatiana Travieso, Jerry Chang, Helen Webster, Lauren E. Williamson, James E. Crowe, Harry B. Greenberg, Huali Wu, Christoph P. Hornik, Katayoun Mansouri, Robert J. Edwards, Victoria Stalls, Priyamvada Acharya, Maria Blasi, Sallie R. Permar

**Affiliations:** a Department of Pathology, Center for Global Health and Diseases, Case Western Reserve University School of Medicine, Cleveland, OH, USA; b Department of Pathology, Duke University School of Medicine, Durham, NC, USA; c Weill Cornell Medicine Department of Pediatrics, Division of Infectious Disease, New York, NY, USA; d Duke Human Vaccine Institute, Duke University School of Medicine, Durham, NC, USA; e Vanderbilt Vaccine Center, Vanderbilt University Medical Center, Nashville, TN, USA; f Department of Pathology, Microbiology and Immunology, Vanderbilt University Medical Center, Nashville, TN, USA; g Department of Pediatrics, Vanderbilt University Medical Center, Vanderbilt, TN, USA; h Departments of Medicine and Microbiology and Immunology, Stanford University School of Medicine, Stanford CA, USA; i The VA Palo Alto Health Care System, Department of Veterans Affairs, Palo Alto, CA, USA; j Department of Pediatrics, Duke University School of Medicine, Durham, NC, USA; k Duke Clinical Research Institute, Duke University School of Medicine, Durham, NC, USA; l Department of Medicine, Duke University School of Medicine, Durham, NC, USA; m Department of Surgery, Duke University School of Medicine, Durham, NC, USA

**Keywords:** Dimeric IgA, Maternal immunity, Neonatal immunity, Passive transfer, Breast milk, Rotavirus

## Abstract

Dimeric IgA (dIgA) is the dominant antibody in many mucosal tissues. It is actively transported onto mucosal surfaces as secretory IgA (sIgA) which plays an integral role in protection against enteric pathogens, particularly in young children. Therapeutic strategies that deliver engineered, potently neutralizing antibodies directly into the infant intestine through breast milk could provide enhanced antimicrobial protection for neonates. Here, we developed a murine model of maternal protective transfer against human rotavirus (RV) using systemic administration of a dimeric IgA monoclonal antibody (mAb). First, we showed that systemically administered dIgA passively transferred into breast milk and the stomach of suckling pups in a dose-dependent manner. Next, we optimized the recombinant production of a potently RV-neutralizing, VP4-specific dIgA (mAb41) antibody. We then demonstrated that systemic administration of dIgA and IgG mAb41 in lactating dams conferred protection from RV-induced diarrhea in suckling pups, with dIgA resulting in lower diarrhea incidence from IgG. Systemic delivery of engineered antimicrobial dIgA mAbs should be considered as an effective strategy for sIgA delivery to the infant gastrointestinal tract via breast milk to increase protection against enteric pathogens.

## Summary

Engineered dimeric IgA infused in lactating dams passively transfers into breast milk and the gastrointestinal tract of suckling neonates and leads to protection against rotavirus-induced diarrhea.

## Introduction

Secretory IgA (sIgA) is the most abundant antibody produced in humans and is important in providing protection against infection at mucosal surfaces like the intestine, oral cavity, upper respiratory tract, and the mammary gland.^1^, ^2^ In mucosal tissues, local plasma cells produce dimeric IgA (dIgA), a multicomponent protein that contains a joining (J) chain connecting two IgA monomers. J chain binds its cognate receptor, polymeric immunoglobulin receptor (pIgR), on the basolateral side of the epithelial cell, triggering internalization and transcytosis of dIgA. Apical proteolytic cleavage of pIgR releases dIgA into the lumen with a portion of pIgR (termed secretory component) still attached; this IgA is referred to as secretory IgA (sIgA). The sIgA antibody mediates protection at the mucosal surface and can have enhanced binding and neutralization capacity against pathogens compared to IgG. For example, sIgA monoclonal antibodies (mAbs) demonstrated enhanced antigen binding and neutralization capacity against enteric and respiratory pathogens when compared to IgG antibodies of the same clone.^3^, ^4^, ^5^, ^6^, ^7^, ^8^ Moreover, systemic delivery of dIgA has been established to be superior to that of monomeric IgA (mIgA) or IgG for delivery into mucosal compartments, such as the intestines in mice^8^ and breast milk in lactating nonhuman primates (NHPs).^9^ Systemic delivery of dIgA mAbs that results in sIgA translocation to the intestines and into breast milk represents a novel therapeutic strategy to reduce infection and transmission of enteric pathogens.

Previous reports demonstrate that direct delivery of sIgA mAbs to the intestine reduced *Salmonella Typhimurium* and *Salmonella enterica* serovar Typhimurium shedding and clinical symptoms in adult and neonatal mouse pups.^8^, ^10^, ^11^ This highlights the potential of using multivalent IgA mAbs in prevention and treatment of enteric diseases. Unlike orally delivered sIgA which would mostly access the intestinal lumen, a systemically delivered dIgA mAb traffics to multiple mucosal sites and could limit pathogen replication both within the lamina propria (as dIgA) and in the lumen (as sIgA). Systemic translocation of dimeric IgA out of the blood and into the intestinal lamina propria and lumen could be an ideal strategy to prevent or treat chronic enteric viral infections such as rotavirus (RV) or norovirus-induced diarrhea in individuals with immunodeficiencies, such as severe combined immunodeficiency^12^, ^13^ and immunodeficiency disorders related to B cell and antibody defects.^14^, ^15^ Additionally systemic delivery of dIgA also can be used in to treat disease in the maternal-neonatal dyad as it traffics to breast milk.^9^ This strategy could reduce replication of enteric pathogens in the maternal intestine and mammary gland ^16^ as well as provide protection to the suckling infant via breast milk.

There is significant evidence suggesting that breast milk provides protection against enteric diseases in suckling offspring. For example, exclusive nursing is associated with decreased diarrhea incidence, prevalence, hospitalizations, and mortality during the first year of life, particularly in developing countries.^17^, ^18^ Indeed, breast milk contains antibodies against enteric pathogens including RV,^19^, ^20^ norovirus,^21^, ^22^ enterotoxigenic *Escherichia coli* (ETEC),^23^ *Shigella*^24^, ^25^, ^26^ and *Vibrio cholerae*.^27^ However, breastmilk antibody titers can be low and vary between women depending on exposure history. Therefore, increasing the amount of potently and broadly neutralizing antibodies in breast milk is likely to decrease enteric infection and disease in infants. Breast milk represents an ideal method for the consistent delivery of mAbs. However, the pharmacokinetics of systemically delivered dIgA antibodies into breast milk and their efficacy against enteric infections is poorly understood.

To further explore this, we tested the hypothesis that maternal systemic delivery of engineered dIgA antibodies would result in delivery of potent sIgA antibodies to the neonatal intestine via breast milk and would protect against a common enteric pathogen RV. In this study, we developed a murine model of maternal systemic passive antibody immunization for transfer into breast milk using a murine dIgA mAb. We demonstrate that following systemic administration of a dIgA mAb in lactating mice, the mAb is rapidly transported into milk and quickly detected in pup stomach content in a dose-dependent manner. We then engineered a murine dIgA version of a potent human RV-neutralizing IgG (mAb41)^28^, ^29^ and optimized it for enhanced production of dIgA antibodies *in vitro*. We show that dams systemically injected with the RV-neutralizing dIgA mAb provided protection to their pups against RV-induced diarrhea. These studies support future clinical assessment of protective transfer of engineered antimicrobial antibodies via maternal passive immunization with dIgA.

## Results

### Maternal systemic administration of dIgA results in antibody transfer into milk of lactating mice and the gastrointestinal tract of their pups

**Fig. 1:**
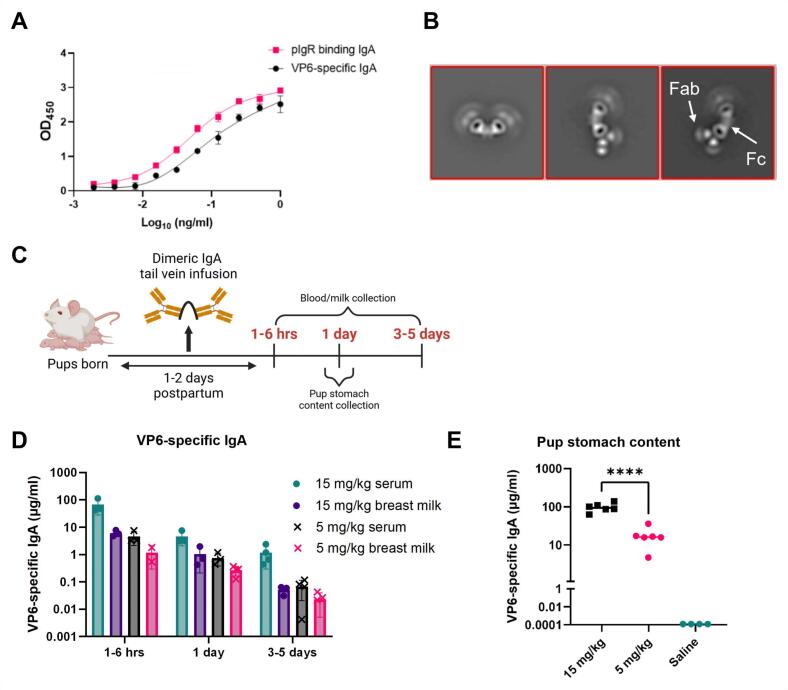
**Dimeric IgA antibodies administered systemically to lactating mice are transferred to milk and the stomach contents of suckling pups**. (A) The rotavirus (RV) VP6-specific, non-neutralizing 7D9 hybridoma derived antibodies bound to RV VP6 (black circles) and polymeric immunoglobulin receptor (pIgR; magenta squares) via ELISA. Data are plotted as mean ± SD of two technical replicates. (B) Negative stain electron microscopy representative images of the hybridoma-purified dimeric IgA (dIgA) 7D9 antibodies. The Fab and Fc regions are indicated. Scale bar represents 10 nm. (C) Schematic of tail vein injections of BALB/c lactating dams given 5 mg/kg or 15 mg/kg dIgA 7D9 at 1 to 2 days postpartum. Blood and milk were collected from dams at 1–6 hrs, 1-, and 3–5-days post injection. A subset of pups (n = 6) per treatment group were sacrificed at 1-day post injection to collect their stomach content. (D) 7D9 antibodies were detected in blood and milk of injected dams via a RV VP6-specific IgA antibody ELISA. The 5 mg/kg (x) and 15 mg/kg (circle) treatment groups are indicated for serum and milk. Data are plotted as mean ± SD of two technical replicates and represent individual mice. (E) 7D9 antibodies were detected in the stomach content of suckling pups via a RV VP6-specific IgA antibody ELISA (15 mg/kg = black squares, 5 mg/kg = pink circles; saline = teal circles). Data are plotted as mean of two technical replicates per individual pup. A significant difference between the compared groups (****p < 0.0001) was determined using an ANOVA. (For interpretation of the references to colour in this figure legend, the reader is referred to the web version of this article.)

To determine whether systemically administered dIgA passively transfers into milk in mice, as previously reported in NHPs,^9^ we first used a RV non-neutralizing dIgA mAb generated from a previously described RV VP6-specific murine dIgA 7D9 hybridoma cell line.^30^ We first confirmed that hybridoma-produced 7D9 dIgA mAbs bound their cognate antigen, the RV capsid protein VP6, and the J-chain receptor pIgR (Fig. 1**A**) demonstrating that 7D9 contains the J-chain needed for pIgR-mediated passive transfer into milk. The dIgA 7D9 purified from hybridoma supernatant was confirmed to contain dimeric antibodies by negative stain electron microscopy (NSEM) (Fig. 1**B**) and size-exclusion chromatography (SEC) (Fig. S1**A**). Dimeric IgA, as determined by a pIgR-binding ELISA, were the most prevalent antibody species present in the purified product (Fig. S1**B**).

To confirm passive transfer of dIgA 7D9 into milk, lactating BALB/c dams were intravenously infused with 5 mg/kg or 15 mg/kg of 7D9 at 1 to 2 days postpartum and plasma and milk were collected at 1–6 hrs, 1-, and 3–5-days after infusion to assess antibody concentrations (Fig. 1**C**). Peak plasma and milk concentrations of 7D9 antibodies, as measured by a VP6-specific IgA ELISA, were observed 1–6 hrs after infusion in both the 5 mg/kg and 15 mg/kg groups (Fig. 1**D**). To confirm transfer of dIgA 7D9 from milk to suckling pups, a subset of pups was sacrificed 1-day post infusion from both the 5 mg/kg (n = 6) and 15 mg/kg (n = 6) litters, and their stomach contents were collected and analyzed for the presence of dIgA 7D9. Dimeric IgA 7D9 was detected in the stomach content of litters born to dams from both 5 mg/kg and 15 mg/kg groups in a dose-dependent manner (Fig. 1**D**). dIgA 7D9 levels precipitously dropped 1-day post infusion in both the serum and milk of lactating dams. Low levels were observed by day 5 post infusion. The kinetics suggest rapid transfer of dIgA 7D9 to mucosal secretions, including milk. These data demonstrate that systemically administered dIgA is efficiently transferred from circulation to milk of mouse dams, like NHPs,^9^ and subsequently into pup stomach contents.

To determine whether passive transfer of dIgA 7D9 into dam milk was pIgR-dependent we systemically injected 5 mg/kg of dIgA 7D9 into pIgR knockout (KO, n = 4) and wildtype (WT, n = 3) mice. At 1 h, 1 day and 3 days post injection, we collected milk and assessed levels of VP6-specific IgA antibodies. We did not observe differences in milk VP6-specific IgA antibodies of pIgR KO and WT mice (Fig. S1**C**). Rogier et al., similarly showed that breast milk levels of IgA were not different in pIgR-/- compared to pIgR-/+ dams at 2–22 days postpartum.^31^ Additionally, Fouda et al. demonstrated passive transfer of monomeric IgA (not containing J chain) into breast milk after intravenous infusion in rhesus macaques.^9^ These previously published results and our data suggest that pIgR-independent transfer of IgA into breast milk is possible.

### Engineering and recombinant production of a RV-neutralizing mouse-human chimeric dIgA

**Fig. 2:**
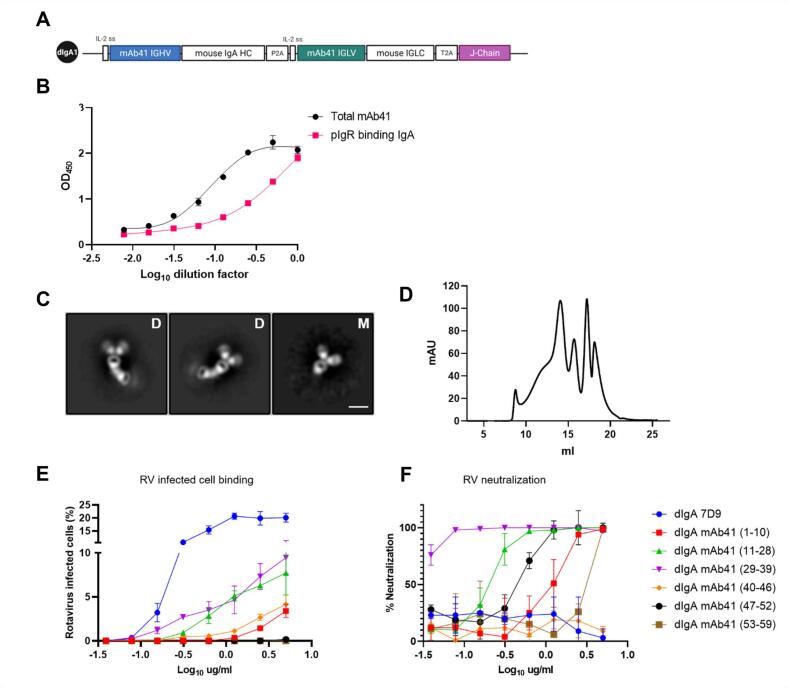
**Recombinant production and characterization of an RV-neutralizing mouse-human chimeric dimeric IgA antibody.** (A) Schematic of the plasmid used to produce the mouse-human chimeric mAb41 dimeric IgA (dIgA). The mAb41 recombined immunoglobulin heavy chain variable region genes (HC), recombined immunoglobulin light chain variable region genes (LC), and the joining chain (J-chain) gene (purple box), are indicated in that order. Schematic created with BioRender. (B) J-chain containing IgA (magenta squares) and total IgA (black circles) antibodies were detected via ELISA. Data are plotted as mean ± SD of two technical replicates. (C) Representative negative stain electron microscopy images of purified dimeric (D) and monomeric (M) mAb41 IgA antibodies. Size exclusion chromatography using a Superose 6 10/300 GL revealed multiple different peaks (Fig. S4). Each of the peaks were fractionated (corresponding fractions 1–10, 11–28, 29–39, 40–46, 47–52 and 52–59) and functionally characterized by rotavirus (RV)-infected cell binding (D) and neutralization assays (E). The non-neutralizing dIgA 7D9 (blue line) was used as a positive control for RV infected cell binding and a negative control for RV neutralization. The IgA isotype control was used as a negative control for both RV infected cell binding and RV neutralization. Data are plotted as mean ± SD of two technical replicates. (For interpretation of the references to colour in this figure legend, the reader is referred to the web version of this article.)

After demonstrating that systemically administered dIgA 7D9 can passively transfer from the periphery to milk using our murine lactation model, we aimed to engineer a potent RV-neutralizing dIgA that could provide protection against RV-induced diarrhea. We constructed a dIgA version of a previously isolated potently RV-neutralizing,VP4-specific mAb, mAb41,^28^, ^29^ by replacing the human IgG1 with the murine IgA constant region and adding the BALB/c J-chain gene on the same open reading frame (Fig. 2**A**). Characterization of the recombinantly produced dIgA mAb41 by ELISA, demonstrated that not all the produced antibodies bound to pIgR (Fig. 2**B**), suggesting that in addition to dIgA, non-dimeric or aggregated IgA species were also present. Indeed, NSEM (Fig. 2**C**) and SEC (Fig. S2) revealed multiple IgA species including monomeric and dimeric (Fig. 2**C**) antibodies. We next fractionated the different IgA species based on size (Fig. S2) and evaluated them for their ability to bind (Fig. 2**D**) and neutralize (Fig. 2**E**) RV. Notably, the fraction suspected to be enriched in dIgA (fraction 29–39) had the greatest RV-binding and neutralization capacity compared to the other isolated fractions (Supplementary Table 1).

**Fig. 3:**
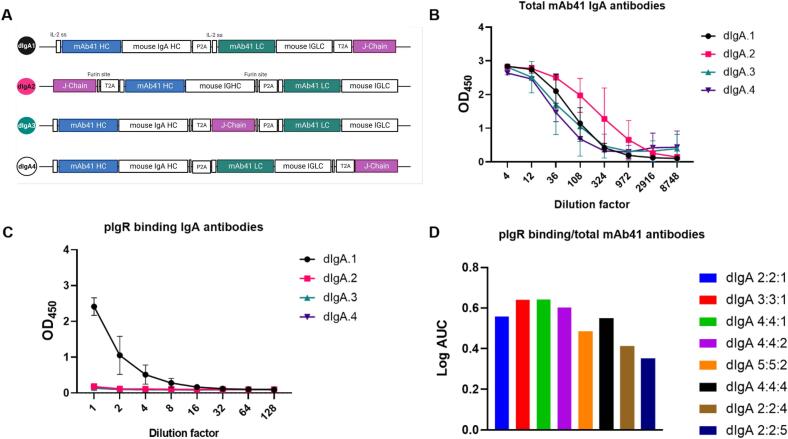
**Antibody chains position and ratio impacts the recombinant production of RV-neutralizing mouse-human chimeric mAb41 dimeric IgA.** (A) Schematic of the different constructs generated to determine if the position of the J-chain gene in the plasmid cassette impacts dimeric IgA (dIgA) production. Illustration created with BioRender. (B) Total IgA antibodies as determined by ELISA. Data are plotted as mean ± SD of experimental duplicates. (C) Polymeric immunoglobulin receptor (pIgR) binding IgA antibodies determined via ELISA. Data are plotted as mean ± SD of experimental duplicates. (D) pIgR IgA antibodies and total mAb41 IgA antibodies were detected via ELISA. Log area under the curve (AUC) was calculated for each co-transfection and graphed as a ratio of pIgR binding IgA antibodies over total mAb41 IgA antibodies.

To generate a more homogenous product and skew antibody production towards increased dimer formation, we designed three additional dIgA constructs, where furin cleavage sites were added before the 2A self-cleaving peptides to enhance cleavage, and the J-chain was placed either in the middle (dIgA.2), at the end (dIgA.3), or at the beginning (dIgA.4) of the ORF (Fig. 3**A**). All constructs produced substantial amounts of total IgA mAb41 antibodies (Fig. 3**B**), however placement of the J-chain gene at the end of the construct (dIgA.1) resulted in the highest yield of pIgR binding IgA antibodies (Fig. 3**C**).

To address whether modulating the ratio between the J-chain and the heavy and light chains resulted in increased dimer formation, we generated three additional plasmids each separately expressing the heavy [H], the light [L] or the J [J] chain genes and compared dimer production following cell transfection with increasing amounts of J-chain plasmid. We observed that the greatest amount of pIgR binding antibodies were produced when using 3:3:1 and 4:4:1 ratio of H:L:J chain plasmids. Additionally, increasing the quantity of J chain plasmid while maintaining constant levels of H and L chain plasmids resulted in a decrease in the production of pIgR-binding antibodies (Fig. 3**D**), concordant with a previous report of recombinant IgA production.^32^.

### Recombinant dIgA mAb41 demonstrates greater RV-binding and neutralization potency than monomeric IgA or IgG1 mAb41 antibodies

**Fig. 4:**
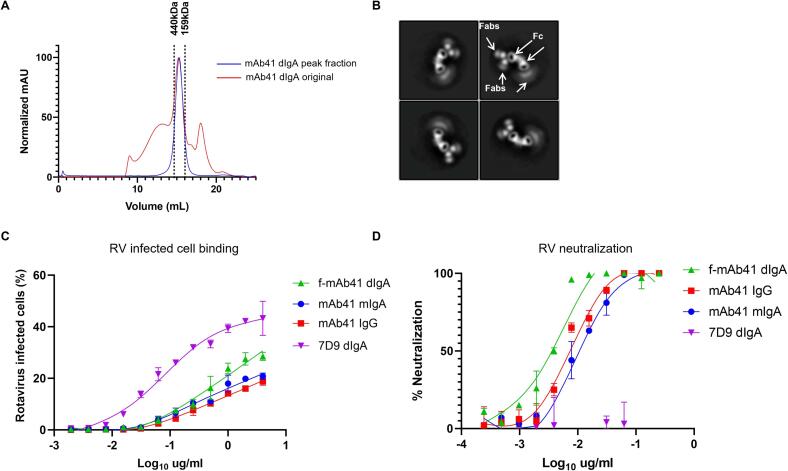
**Recombinant mAb41 dimeric IgA antibodies demonstrate greater rotavirus (RV) binding and neutralization potency than mAb41 monomeric IgA or IgG antibodies**. (A) Size exclusion chromatography by Superose 6 Increase 10/300 GL column in 1XPBS of mAb41 dimeric IgA (dIgA) antibodies at a flow rate of 0.75 ml/min. Molecular weight markers are listed above the dashed lines at 440 kDa and 158 kDa, respectively. (B) Negative stain electron microscopy of the purified dimeric antibodies. Fab and Fc regions are indicated respectively by white arrows. (C) RV infected cell binding assay. Data are plotted as mean ± SD of two technical replicates. (D) RV neutralization assay. Data are plotted as mean ± SD of two technical replicates.

To exclude functional contributions from aggregated dimeric/polymeric or monomeric IgA antibodies, recombinantly produced dIgA mAb41 was fractionated by SEC (Fig. 4**A**) and the average molecular weight of 300 kDa was selected as fractionated dIgA mAb41 (f-dIgA mAb41), which is consistent with the expected mass of dIgA at approximately 335 kDa. NSEM demonstrated that the f-dIgA mAb41 product contained only dimeric antibodies (Fig. 4**B**)**.** The f-mAb41 dIgA product was then assessed for functional capacity in comparison with mIgA mAb41 and IgG1 mAb41. f-dIgA mAb41 demonstrated greater RV-binding capacity and neutralization activity (AUC = 193.4; IC_90_ = 7.1 ng/ml) compared to mIgA (AUC = 136.3; IC_90_ = 44.8 ng/ml) or IgG1 (AUC = 155.3; IC_90_ = 33.1 ng/ml) (Fig. 4**C and 4D,** Supplementary Table 1). This difference in neutralization activity was greater when IC_90_ values were normalized for antibody molecular weight, resulting in a 42-fold reduction in IC_90_ value for f-dIgA mAb41 (21.2 pM) compared to mIgA (298.3 pM) and IgG (220.5 pM).

### Pharmacokinetics (PK) of intravenous dIgA 7D9 infusion in milk of lactating BALB/c dams

To determine the optimal dose of dIgA for systemic maternal infusion and breast milk transfer using our model dIgA 7D9 antibody, we used an Empirical Bayesian estimate of individual PK parameters in both plasma and milk from dIgA 7D9-infused dams (Fig. 1**D**). Antibody level data were used in simulation for multiple intravenous doses including 5 mg/kg, 10 mg/kg, and 15 mg/kg (Fig. S3). To estimate the antibody concentrations in milk, dIgA 7D9 concentrations were simulated at 24 to 192 hrs in 24-hr intervals. Dosing intervals of 1 to 3 days were explored. Using a 1-day dosing interval, concentrations of dIgA 7D9 remained stable in milk up to 8 days after the first dose (Fig. S3**A**). However, with the 2- (Fig. S3**B**) and 3-day (Fig. S3**C**) dosing intervals, dIgA 7D9 concentrations in milk dropped by day 2 post-infusion and continued to decrease without an additional dose. The intercompartmental clearance from plasma to milk was 0.11 mL/h and the elimination half-life was 13.55 (6.13 – 18.90) hrs. The observed elimination half-life of dIgA 7D9 is like that of dIgA reported in other species (<1 day to ∼ 4 days), including mice and rhesus macaques.^32^, ^33^ Interestingly, systemic infusion of 5 mg/kg IgG1 mAb41 antibodies in lactating mice yielded similar kinetics with a rapid decline in antibody levels in dam milk, which were cleared by day 8 post infusion (Fig. S4).

## Maternal passive immunization with a PK-optimized dose of systemic dIgA mAb41 protects against RV-induced diarrhea in suckling pups

**Fig. 5:**
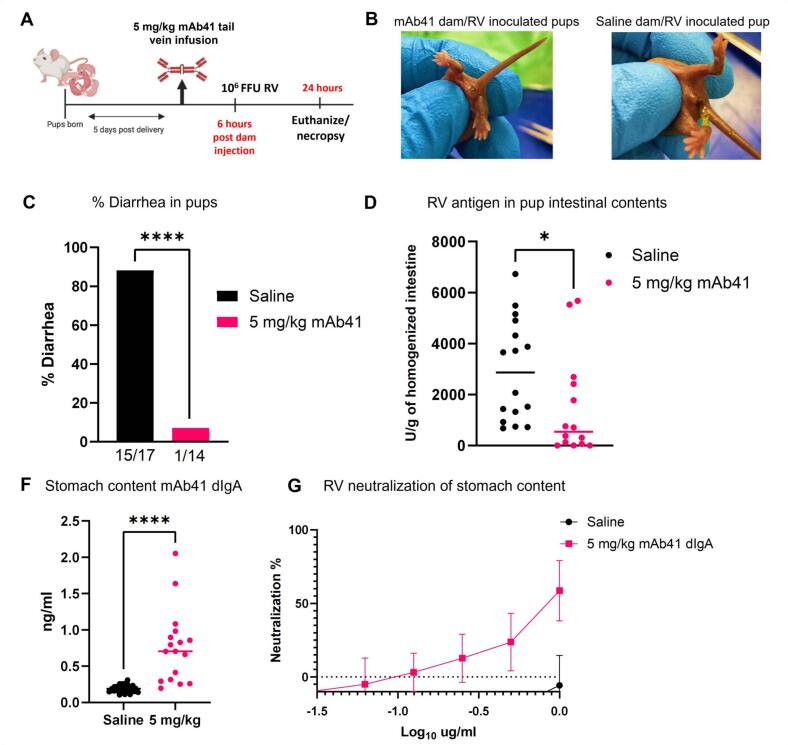
**Passive maternal immunization with systemic mAb41 dIgA protects against rotavirus (RV)-induced diarrhea in suckling pups**. (A) Schematic of tail vein injections of BALB/c lactating dams with 5 mg/kg f-dIgA mAb41 antibodies at 5 days postpartum. Pups were orally inoculated with 1 × 10^6^ FFU of RV (Wa strain) at 1–2 hrs post dam injection and euthanized 24 hrs later. Schematic created with BioRender. (B) Representative image of RV inoculated suckling pups from f-dIgA mAb41 infused dams, which excreted urine or hard stool upon abdomen gentle palpation. (C) Representative image of RV inoculated suckling pups from saline infused dams, which excreted yellow, liquid and/or stick stool after gentle abdomen palpation. (D) Diarrhea was reported as % of animals with clinical symptoms upon gentle abdomen palpation in each treatment group (5 mg/kg = blue; saline = red). The number of animals with diarrhea out of the total number of animals are reported at the top of each bar graph. (E) RV antigen in homogenized intestinal tissue was detected via a commercial RV antigen binding ELISA. Significant differences between the compared groups were determined using a Mann-Whitney *U* test (**p < 0.05). (F) mAb41 antibodies were detected in stomach content of suckling pups using an mAb41 anti-idiotypic IgA antibody ELISA. Significant differences between the compared groups were determined using a Mann-Whitney *U* test (***p < 0.001). (G) Stomach content was assessed for RV neutralization at different dilutions and plotted as neutralization % in n = 6 pups per treatment group (5 mg/kg = blue; saline = red). Data from C-F are from 3 litters per treatment group (for litter distribution see Supplementary Table 1 and Fig. S6). Data are plotted as the mean ± SD of two technical replicates. (For interpretation of the references to colour in this figure legend, the reader is referred to the web version of this article.)

To determine if passive transfer of dIgA mAb41 in breast milk results in protection from RV induced diarrhea in suckling neonates, we developed a lactating dam dIgA-infusion and RV challenge model using 129sv mice dams and their pups. We chose the 129sv mouse strain as 129sv pups develop detectable diarrhea after oral inoculation with human RV.^28^ Lactating 129sv dams were injected in the tail vein with 5 mg/kg of f-dIgA mAb41 at 4 to 6 days postpartum (Fig. 5**A**). Due to the short half-life of dIgA in milk, as determined by our PK analysis (Fig. S3) and to maximize the amount of f-dIgA mAb41 in the gastrointestinal tract of suckling pups at the time of RV inoculation, pups were inoculated between 1–2 hrs post dam injection with 1 × 10^6^ Fluorescent Focus Units (FFU) of RV Wa strain. Litters born to dams passively immunized with 5 mg/kg dIgA mAb41 had lower incidence of diarrhea (1 of 14, 7.1 %) upon gentle abdomen palpation (Fig. 5**B and 5D**) compared to litters born to saline-immunized dams (15 of 17, 88 %) (Fig. 5**C,D**). Consistently, significantly lower RV antigen in the intestine was observed in pups born to dIgA mAb41-immunized mothers compared to pups of saline-injected mothers (Fig. 5**E**). Moreover, dIgA mAb41 was detected in the stomach contents of the suckling pups (Fig. 5**F**), which also exhibited RV neutralization capacity at the lowest dilution measured (Fig. 5**G**) without compromising viability of the cell monolayer (Fig. S5). Thus, dIgA mAb41 passively transferred to suckling pups through the milk of lactating dams protected against RV-induced diarrhea.

**Fig. 6:**
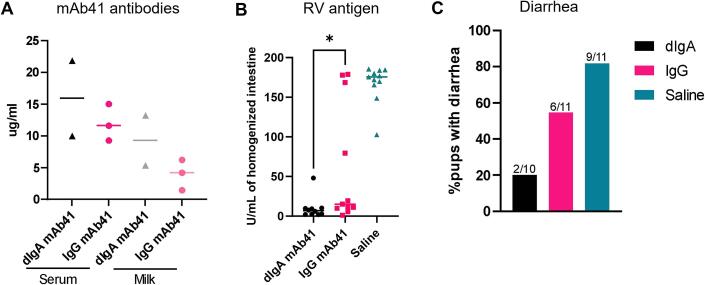
**Passive immunization with dimeric IgA mAb41 decreases intestinal rotavirus antigen and diarrhea scores in suckling pups.** (A) Concentrations of dIgA and IgG mAb41 in dams’ serum and milk were determined by mAb41 anti-idiotypic antibody ELISA. (B) RV antigen in pups homogenized intestinal tissue was detected via a commercial RV antigen binding ELISA. Significant differences between the compared groups were determined using a one-way ANOVA (*p < 0.05). (C) Diarrhea was reported as the percentage of animals with diarrhea at day 1 post-challenge in each treatment group. The number of animals with diarrhea out of the total number of animals are reported at the top of each bar graph. Data from B-C are from 3 L per treatment group (for litter distribution see Supplementary Table 1 and Fig. S6). Data are plotted as the mean ± SD of two technical replicates.

Finally, we sought to determine whether passive transfer of IgG mAb41 into breast milk following systemic administration provides protection against RV-induced diarrhea. Lactating 129sv dams were tail vein injected with 5 mg/kg of f-dIgA mAb41, IgG1 mAb41, or saline at 4 to 6 days postpartum (Fig. 6**A**). Concentrations of IgG and dIgA mAb41 were similar in dam serum while concentrations of IgG mAb41 in milk were lower, although not significantly, compared to that of dIgA mAb41 (Fig. 6**A**). Pups were inoculated with 1 × 10^6^ FFU of RV Wa strain between 1 and 2 hrs post dam injection. Litters born to dIgA mAb41-immunized dams had significantly decreased RV antigen in feces compared to IgG mAb41-immunized dams, although this difference was primarily driven by a subset of pups with high RV antigen levels (Fig. 6**B**). Litters born to dams injected with 5 mg/kg f-dIgA mAb41 had also lower incidence of diarrhea (2 of 10, 20 %) upon gentle abdomen palpation compared to litters born to IgG1 mAb41 (6 of 11, 54 %) or saline-immunized dams (9 of 11, 82 %) (Fig. 6**C**).

## Discussion

In this study, we sought to evaluate whether maternal systemic administration of an engineered dIgA mAb could traffic to breast milk and mediate protection against RV-mediated disease in the suckling neonates. Using a mouse lactation model, we demonstrated that a systemically administered dIgA passively transferred into dams’ breast milk, as previously established in NHPs,^9^ and was delivered to the gastrointestinal tract of suckling pups. We then investigated the half-life of an engineered, potent RV-neutralizing dIgA (mAb41 dIgA). After systemic infusion, the elimination half-life was short [13.55 (6.13 – 18.90) hrs] compared to what is reported for circulating IgG (15–30 days depending on the subclass) ^34^ and was consistent with previous reports.^32^ To compare the levels of systemically administered dIgA to that of IgG in mouse milk, we also systemically infused lactating mice with 5 mg/kg of IgG1 mAb41. Similarly, to dIgA 7D9, mAb41 IgG1 levels in milk became undetectable by day 8 post-infusion. Notably, days to clearance of dIgA and IgG1 mAb41 in mouse milk observed in this study were much shorter than what is observed in milk after dIgA and IgG1 infusion in rhesus macaques, as levels were still detectable after 42 days post infusion in 2 out of 3 and 4 out of 4 lactating dams, respectively.^9^ The shorter half-life in mice compared to rhesus macaques may be due to hepatobiliary transport of IgA into bile and ultimately into the intestine.^35^, ^36^ These data also highlight that strategies to extend the half-life of IgA, including recombinant viral vectors and mRNA delivery, are warranted.

Exploration of the therapeutic potential of neutralizing dIgA *in vivo* has been hampered by the difficulties in production and purification of dIgA at desired antibody quantities.^37^, ^38^ For example, ours and others^32^, ^39^, ^40^ have reported multiple species of IgA, including polymeric, dimeric, and monomeric forms as well as aggregated IgA, in dimeric IgA recombinant preparations. To be confident we characterized only dIgA and not other IgA species *in vivo*, we purified our dIgA recombinant preparations by size exclusion chromatography. This caused a significant loss of product that required more recombinant dIgA transfection preparations than if we weren’t concerned with preparation purity. Therefore, to optimize the production of a potent RV-neutralizing dIgA (mAb41) for greater dIgA yields, we generated several plasmid constructs. We observed that the construct where the J-chain gene was placed after the heavy and light chain genes produced the highest level of dIgA following recombinant production. This suggests that spatiotemporal production of the IgA heavy, light and J-chain in the cell influences dimerization and therefore, dIgA yield. We also observed that decreasing the amount of J-chain DNA relative to heavy and light chain DNA, resulted in higher levels of dIgA. These results are not surprising, given that each dimer comprises two IgA monomers linked together by a single J-chain.

Functional characterization of the newly generated mAb41 dIgA demonstrated that the dimer had higher neutralization potency compared to both IgA and IgG1 monomers which is likely due to the higher number of available binding sites present on dimers compared to monomers. Isotype-specific mAb protection has been previously demonstrated in both *in vitro* and *in vivo* studies. Mice were better protected from influenza infection in the nasopharynx after systemic administration of anti-influenza polymeric IgA antibodies compared to IgG.^5^, ^6^, ^7^ Interestingly, recombinantly produced poliovirus-specific antibodies had similar neutralization activity whether they were produced as mIgA, dIgA or IgG,^41^ suggesting that isotype-specific differences in functional capacity may be pathogen and even epitope specific.

Neutralizing antibodies against the external RV proteins VP4 and VP7 play an important role in protective immunity against RV infection.^42^, ^43^, ^44^, ^45^ Using the 129sv mouse model of human RV challenge^28^ we showed that a systemically administered RV-neutralizing, VP4-specific dIgA antibody passively transferred into breast milk and protected suckling neonates from RV-induced diarrhea. Additionally, systemic mAb41 IgA infusion offered increased protection compared to an IgG1 antibody of the same clone. Nevertheless, 45.4 % of mAb41 IgG infused mice were still protected from RV-induced diarrhea, suggesting that increasing the concentration of neutralizing IgGs in breastmilk is another potential strategy to reduce the burden of enteric diseases in infants. However, a limitation of our mouse model is that human rotavirus A did not induce diarrhea beyond 1–2 days post-inoculation, restricting the ability to perform longer-term analyses. Further investigation using alternative models, such as gnotobiotic piglets,^46^ is warranted to enable extended studies of disease progression and intervention efficacy.

While passive antibody transfer studies via breast milk have yet to be performed in human infants, oral delivery of recombinant mAbs has been explored. Interestingly, while orally fed palivizumab (anti-RSV IgG1 mAb) was not stable across the infant gastrointestinal tract,^46^ natural anti-RSV IgG and IgA from breast milk were stable through all phases of simulated infant digestion.^47^ This finding suggests that delivering pathogen-specific IgG and IgA via breast milk may be a more effective strategy than direct infant administration due to increased stabilization of breast milk antibodies in the gastrointestinal tract.

Maternal systemic delivery of dIgA offers a wide range of therapeutic potential against infection. A systemically delivered recombinant dIgA in lactating mothers would target both the mammary gland/breast milk and the maternal gut, helping to limit maternal RV infection and thereby reducing the RV environmental load in the household and community. Additionally, and as mentioned above, enteric infections like RV can cause chronic diarrhea in immunodeficient children and adults. Therefore, systemic delivery of potently neutralizing mAbs that traffic to the intestine in these vulnerable groups could limit infection and resolve chronic disease. DIgA is also able to traffic to the respiratory tract in small animal models,^5^, ^6^ suggesting that systemic delivery of dIgA mAbs can also alleviate infection and disease from respiratory pathogens.

Despite being a promising approach, intravenous delivery of recombinant antibodies can be challenging especially in low- and middle-income countries. Therefore, the long-term goal of this approach is to use a delivery platform such as recombinant viral vectors or mRNA that can express dimeric IgA *in vivo* long-term, circumventing the need for repeated injection. In summary, our data support the development of passive immunization strategies with neutralizing antibodies in lactating women to protect against neonatal enteric pathogens, as well as breast milk-transmitted pathogens like HIV, particularly in highly vulnerable populations. Our results will help guide the development of novel maternal immunization strategies, which may leverage passive transfer of potent anti-microbial IgG and dIgA into breastmilk to decrease infant morbidity and mortality against enteric pathogens.

## Methods

### Cells and viruses

African Green Monkey kidney epithelial cell line MA104 (CRL-2378.1) was obtained from American Type Culture Collection (ATCC) and cultured in MEM-alpha (Life Technologies) supplemented with 10 % fetal bovine serum (FBS), 50 Units/mL of penicillin and 50 µg/ml of streptomycin (Invitrogen). RV strain A (Wa) (ATCC) was propagated in MA104 cells as previously described.^48^ .

### RV quantification

RV was quantified using a fluorescence focus assay.^48^ In brief, RV was activated with 10 µg/ml of trypsin for 1 hr in a 37 °C water bath. Serially diluted virus was added to confluent MA104 cells and incubated for 1 hr at 37 °C. Inoculum was removed and growth medium including DMEM (Life Technologies), 5 % FBS, 50 Units/mL of penicillin and 50 µg/mL of streptomycin (Invitrogen) was added. Infected cells were then incubated at 37 °C for 12 to 18 hrs. Medium was removed from the plates and fixed with 10 % formalin in neutral buffered saline for 20 min. Wells were then washed with 2 % FBS and cells were permeated with 0.5 % Triton-X in PBS for an additional 15 min. Wells were washed twice and 7D9 (VP6-specific, murine IgA antibody) was added at 10 µg/ml in 2 % FBS as the primary detection antibody for 1 hr at room temperature (RT). Cells were washed twice, and an anti-murine IgA antibody conjugated to FITC (1:100; Southern Biotec) was added to wells for 1 hr at RT. Cells were washed four times with wash solution and DRAQ5 nuclear stain (Fisher Scientific) was added to cells at 1:2000 dilution. Cells were washed once with PBS and resuspended in 10 µl of PBS. Infection was quantified in each well by automated cell counting software using a Cellomics Arrayscan VTI HCS instrument at × 10 magnification. Subsequently, the percent of infected cells was determined as FITC^+^DRAQ5^+^ cells.

### 7D9 antibody production

The 7D9 hybridoma line was cultured in ClonaCell™-HY Medium E (STEMCELL Technologies) prior to antibody production. To produce large quantities of 7D9, cells were resuspended in Hybridoma Serum Free Medium (Fisher Scientific) and seeded in the cell compartment of a bioreactor, with Medium E providing nutrients from the medium compartment. Antibody was harvested from the cell supernatant after 5 to 7 days post inoculation and purified using Protein L Sepharose beads (Thermo Fisher Scientific).

### Construction of mAb41 plasmids and antibody production

To generate the human-mouse chimeric mAb41 IgA and IgG, we took the variable domain sequences of a previously isolated human anti-RV VP4 specific neutralizing mAb (*i.e*., mAb#41),^28^ attached it to the murine IgA and IgG constant regions (accession numbers: AB644393.1, JQ048937.1, KT336476.1, JQ048937.1), respectively, and cloned it into the pcDNA3.1 expression vector. A third plasmid encoding the BALB/c J-chain sequence (accession number: AB664392.1) was also generated. The heavy, light and J chain of mAb41 IgA were also cloned into a single open reading frame, and in different orientations as shown in Fig. 4. The 2A self-cleaving peptide technology was used to express both the heavy, light and J chain genes from a single open reading frame. Antibodies were produced by transient transfection of human epithelium kidney 293 T Lenti-X cells (Clontech Laboratories, Mountain View, CA) using the JetPrime transfection kit (Polyplus Transfection Illkirch, France) following the manufacturer's recommendations. Different amounts of each plasmid were transfected as shown in Fig. 4. Antibodies were harvested from cell supernatants at 4 to 5 days post-transfection and purified using CaptureSelect™ LC-lambda (mouse) Affinity Matrix (Thermo Fisher Scientific).

### Dimeric IgA characterization and purification

Dimeric antibodies (7D9 and mAb41) were characterized and fractionated by size exclusion chromatography using a Superose 6 10/300 GL on an AKTA liquid chromatography system and concentrated on AmiconUltra 100 k spin columns (Millipore).

### Negative-stain electron microscopy (NSEM)

Antibodies were diluted to 100 μg/ml final concentration with buffer containing 10 mM NaCl, 20 mM HEPES buffer, pH 7.4, 5 % glycerol and 7.5 mM glutaraldehyde. After 5-minute incubation, excess glutaraldehyde was quenched by adding sufficient 1 M Tris stock for a final Tris 75 mM for 5 mins; then samples were stained with 2 % uranyl formate. Images were obtained with a Philips 420 electron microscope operated at 120 kV, at 82,000 × magnification and a 4.02 A˚ pixel size. RELION 3.0^49^ was used for CTF correction, automatic particle picking and 2D class averaging of the single-particle images.

### Animals

All animal procedures were conducted in strict accordance with the guidelines outlined in the Guide for the Care and Use of Laboratory Animals with proper enrichment and were approved by the Duke University IACUC under protocol A178-21–08. Pups were monitored for > 20 % weight loss as human euthanasia criteria.

Timed pregnant BALB/c and 129sv mice were obtained from Charles River laboratories and Taconic Biosciences, respectively. The pIgR knockout mice were a generous gift of the pIgR knockout mice. Upon arrival, all mice were maintained in a pathogen-free animal facility under a standard 12 hr light/12 h dark cycle at RT with access to food and water *ad libitum*. Timed pregnant mice received a supplemental nutritional gel to decrease risk of pup savaging. For IV injections of recombinant dIgA and IgG mAbs, animals were restrained using a mouse tail vein restrainer. For mouse milking, dams were separated from their pups for at least 2 hrs to allow milk accumulation while pups were kept warm on a heating pad. Dams were administered 2 IU/kg of oxytocin via intraperitoneal (IP) injection. The mammary area was wiped with sterile alcohol prep pad before manually expressing the teat with thumb and forefinger to gently massage the mammary tissue in an upward motion until a visible bead of milk formed at the base of the teat. A sterile pipet tip was used to gently pull the milk into the tip. All teats were milked two times. Milk was diluted 1:4 with PBS and filtered with 0.22 μm Spin-x centrifugal filters (Costar) at 4 °C at 15,000 x g for 30 min. The Spin-x filter separated the lipid portion of the milk from the liquid whey portion, and the liquid whey portion was stored in −20 °C. Blood samples were collected from the facial vein (submandibular). The blood was allowed to clot at ambient temperature. Clotted blood samples were maintained at RT and centrifuged for 6,000 RPM for 15 min. The serum was separated from the blood and stored at −20 °C.

For RV infection, neonatal 129sv mice (5 days old) were orally gavaged with a minimum of 1 × 10^6^ FFU of RV Wa. Pup stomach contents and intestines were collected and homogenized in 500 µm PBS using a TissueLyzer II (Qiagen) for 5 min at 50 Hz with a stainless-steel ball added as a pulverizer, in addition to manual pulverization using a pipet tip. Pulverized stomach content and intestinal tissue were transferred to a new microcentrifuge tube and spun for 10 min at 3,000 RPM. Supernatants were collected and then filtered via 0.22-µm Spin-x centrifugal filter tubes by centrifugation at 18,000 × *g* for 20 mins at 4 °C. A protease cocktail (1X) (Fisher Scientific) was added and samples were stored at −20 °C until further analysis.

### VP6 binding IgA antibody ELISA

Recombinant VP6 protein (head domain; residues 147–339 of full-length VP6) was expressed in *E. coli* and purified through affinity chromatography using a Ni-NTA column and size-exclusion chromatography using a Superdex 200 10/300 GL column as previously described.^50^ Nunc® Maxisorp™ 384-well plates were coated with 3 µg/ml of recombinant VP6 protein diluted in coating solution concentrate (Seracare) overnight at 4 °C. Plates were washed one time (PBS, 0.5 % Tween-20) and incubated for 2 hrs with blocking solution (PBS, 4 % whey protein, 15 % goat serum, 0.5 % Tween-20). Antibodies and biological samples were diluted in blocking solution and added to wells in duplicate for 1 hr. Plates were then washed twice and incubated for 1 hr with an HRP-conjugated, goat anti-mouse IgA antibody (Southern Biotech) at a 1:5000 dilution. After 4 washes, SureBlue Reserve TMB Microwell Peroxidase Substrate (KPL) was added to the wells for 10 mins, and the reaction was stopped by addition of 1 % HCl solution. Plates were read at 450 nm. OD values within the linear range of a standard curve were used to interpolate the concentration of VP6-binding IgA antibodies in the transfection products. The standard curve was generated by serial dilutions of dIgA 7D9.

### pIgR binding IgA antibody ELISA

J-chain containing IgA antibodies were measured by pIgR binding ELISA. Nunc® Maxisorp™ 384-well plates were coated with 6 µg/ml of recombinant mouse pIgR protein (R&D Systems) diluted in coating solution concentrate (Seracare) overnight at 4 °C. The ELISA assay was completed as described above. OD values within the linear range of a standard curve were used to interpolate the concentration of pIgR-binding IgA antibodies in the transfection products. The standard curve was generated by serial dilutions of dIgA 7D9.

### mAb41 anti-idiotypic antibody ELISA

Nunc® Maxisorp™ 384-well plates were coated with 1 µg/ml of an mAb41 anti-idiotypic antibody (Biogenes GmbH) diluted in coating solution (Seracare) overnight at 4 °C. The ELISA assay was completed as described above for detecting IgA mAb41 antibodies. For detecting IgG mAb41 antibodies, a biotinylated mAb41 anti-idiotypic antibody was used as the secondary antibody at 2 μg/ml. A streptavidin-HRP conjugate (Abcam, 1:10,000) was used to detect the secondary antibody prior to development using the SureBlue Reserve TMB Microwell Peroxidase Substrate (KPL) and1% HCl stop solution as described above. OD values within the linear range of a standard curve were used to interpolate the concentration of pIgR-binding IgA antibodies in the transfection products. The standard curve was generated by serial dilutions of mIgA or IgG mAb41 antibodies.

### RV infected cell binding assay

MA104 cells were seeded into 96-well plates and incubated until confluent (3–4 days) at 37 °C and 5 % CO_2_. RV Wa was thawed at RT and activated with 10 µg/ml of trypsin for 30 mins at 37 °C. RV was added to cells at MOI 2 and incubated at 37 °C and 5 % CO_2_ for 20 to 22 hrs. Cells were fixed with 10 % neutral buffered formalin for 20 mins. Cells were washed once with wash solution (2 % FBS in PBS). To permeate cell membranes, 0.5 % Triton-X in PBS was added to cells for 15 mins. Cells were washed twice and 7D9 added to all wells at 10 µg/ml and incubated for 1 hr in the dark at RT. Cells were washed twice with wash solution and an anti-mouse IgA FITC secondary (Abcam) was added at 1:100 dilution and incubated for 1 hr in the dark at RT. Cells were washed four times with wash solution and DRAQ5 nuclear stain (Fisher Scientific) was added to cells at 1:2000 dilution. Cells were washed once with PBS and resuspended in 10 µl of PBS. Infection was quantified in each well by automated cell counting software using a Cellomics Arrayscan VTI HCS instrument at × 10 magnification. Subsequently, the percent infected cells were determined as FITC^+^DRAQ5^+^ cells. 7D9 was used as a positive RV infected cell binding control and a commercial IgA isotype control (Invitrogen; 14–4762-81) was used as a negative RV infected cell binding control.

### RV neutralization assay

MA104 cells were seeded into 96-well plates and incubated until confluent (3–4 days) at 37 °C and 5 % CO_2_. RV Wa was thawed at RT and activated with 10 µg/ml of trypsin for 30 mins at 37 °C. Serial dilutions of mAbs or homogenized stomach contents were incubated with RV Wa (MOI = 4) in 50 μl for 1.5 hrs at 37 °C. The virus/mAb or virus/plasma dilutions were then added in duplicate to wells containing MA104 cells and incubated at 37 °C for 20 to 22 hrs. Cells were fixed with 10 % neutral buffered formalin for 20 mins. Cells were washed once with wash solution (2 % FBS in PBS). To permeate cell membranes, 0.5 % Triton-X in PBS was added to cells for 15 min. Cells were washed twice and 7D9 added to all wells at 10 µg/ml and incubated for one hr in the dark at RT. Cells were washed twice with wash solution and anti-mouse IgA FITC secondary (Abcam) was added at 1:100 dilution and incubated for 1 hr in the dark at RT. Cells were washed four times with wash solution and DRAQ5 nuclear stain (Fisher Scientific) was added to cells at 1:2000 dilution. Cells were washed once with PBS and resuspended in 10 µl of PBS. Infection was quantified in each well by automated cell counting software using a Cellomics Arrayscan VTI HCS instrument at × 10 magnification. Subsequently, the ID_50_ was calculated as the sample dilution that caused a 50 % reduction in the number of infected cells compared with wells treated with virus only using the Reed and Muench method. 7D9 and a commercial IgA isotype control (Invitrogen; 14–4762-81) were used as negative RV neutralization controls.

*RV antigen ELISA.* An EDI fecal RV antigen ELISA kit was used according to the manufacturer’s protocol. In brief, 100 µl aliquot of the homogenized intestinal samples were diluted in kit diluent and added in equal volumes to duplicate wells. A set of standards was included (0, 1.9, 5.6, 16.7, 50, 150 U/mL). Samples were incubated for 1 hr at RT. Wells were washed 5 times with washing buffer and incubated with 100 µl of tracer antibody for 30 mins at RT. The wells were washed and 100 µl of the antibody substrate was added. Samples were incubated in the dark for up to 15 mins and 100 µl of stop solution was added to stop the reaction. The absorbance readings were generated at 450 nm. A standard curve was plotted and the antigen concentration (U/mL) in the samples was calculated from the curve.

### Statistical analysis

Statistical analyses were performed using Prism software v9.4.1 (GraphPad, San Diego, CA, USDA). Student’s *t* test for parametric results or the non-parametric Mann–Whitney test was performed to compare two groups. To ensure unbiased allocation, time pregnant dams arriving from the supplier were randomly assigned to treatment groups using R statistical software. The comparison between three or more groups was performed by one-way analysis of variance, followed by multiple comparisons according to Tukey’s test for parametric data or the Kruskal–Wallis test, followed by Dunn’s multiple comparisons test for non-parametric data. The sample size for the rotavirus infection experiments was determined based on the experimental design, involving two groups: saline-injected dams and dimeric IgA-injected dams. Unless otherwise stated, data are expressed as mean + SD. Differences were considered statistically significant at *p* < 0.05.

## Author contributions

S.N.L. contributed to study design, analyzed the data, and wrote the manuscript. S.N.L., J.T., J.C., T.T., H.W., C.E.O., L.W., J.C., H.G. performed experiments, including antibodies production, ELISA, neutralization assays and *in vivo* studies. W.H. and H.C. performed the pharmacokinetics analysis. R.E. and K.M. performed the negative stain electron microscopy. V.S. and P.A. performed size exclusion chromatography and molecular weight determination. M.B. and S.R.P. conceived the study, oversaw the planning and direction of the project including analysis and interpretation of the data and editing of the manuscript. All authors read, revised, and approved the final manuscript.

## CRediT authorship contribution statement

**Stephanie N. Langel:** Writing – review & editing, Writing – original draft, Validation, Supervision, Methodology, Investigation, Formal analysis, Data curation. **Claire E. Otero:** Writing – review & editing, Methodology, Formal analysis, Data curation. **Justin T. Steppe:** Methodology, Formal analysis, Data curation. **Caitlin A. Williams:** Writing – review & editing, Formal analysis, Data curation. **Tatiana Travieso:** Writing – review & editing, Validation, Investigation, Data curation. **Jerry Chang:** Writing – review & editing, Formal analysis, Data curation. **Helen Webster:** Writing – review & editing, Formal analysis, Data curation. **Lauren E. Williamson:** Writing – review & editing, Formal analysis, Data curation. **James E. Crowe:** . **Harry B. Greenberg:** Writing – review & editing, Methodology. **Huali Wu:** Writing – review & editing, Formal analysis, Data curation. **Christoph P. Hornik:** Writing – review & editing, Supervision, Methodology. **Katayoun Mansouri:** Writing – review & editing, Formal analysis, Data curation. **Robert J. Edwards:** Writing – review & editing, Supervision, Methodology. **Victoria Stalls:** Writing – review & editing, Formal analysis, Data curation. **Priyamvada Acharya:** Writing – review & editing, Supervision, Methodology. **Maria Blasi:** Writing – review & editing, Supervision, Resources, Methodology, Investigation, Funding acquisition, Formal analysis, Data curation, Conceptualization. **Sallie R. Permar:** Writing – review & editing, Supervision, Resources, Project administration, Methodology, Investigation, Funding acquisition, Formal analysis, Data curation, Conceptualization.

## Declaration of competing interest

The authors declare the following financial interests/personal relationships which may be considered as potential competing interests: [J.E.C. has served as a consultant for Luna Biologics, is a member of the Scientific Advisory Board of Meissa Vaccines and is Founder of IDBiologics. The Crowe laboratory at Vanderbilt University Medical Center has received unrelated sponsored research agreements from Takeda Vaccines, IDBiologics and AstraZeneca. S.R.P. provides individual consulting services to Moderna, Merck, Dynavax, GSK, and Pfizer on CMV vaccines. Merck Vaccines and Moderna have provided grants and contracts for S.R.P. sponsored programs.].
