# Supplementary material for: Breast milk delivery of an engineered dimeric IgA protects neonates against rotavirus
Source: Mucosal Immunol. 2025 Apr;18(2):441–52. doi: 10.1016/j.mucimm.2025.01.002 (PMC11982437; doi:10.1016/j.mucimm.2025.01.002)
Supplement: Supplementary Data 1 [file mmc1.pdf]

**Supplemental information**

# Breast milk delivery of an engineered dimeric IgA protects neonates against rotavirus

Langel SN, Otero CE, Steppe JT, Williams CA, Travieso T, Chang J, Webster H, Williamson LE, Crowe JE Jr, Greenberg H, Wu H, Hornik C, Mansouri K, Edwards RJ, Stalls V, Acharya P, Blasi M, Permar SR

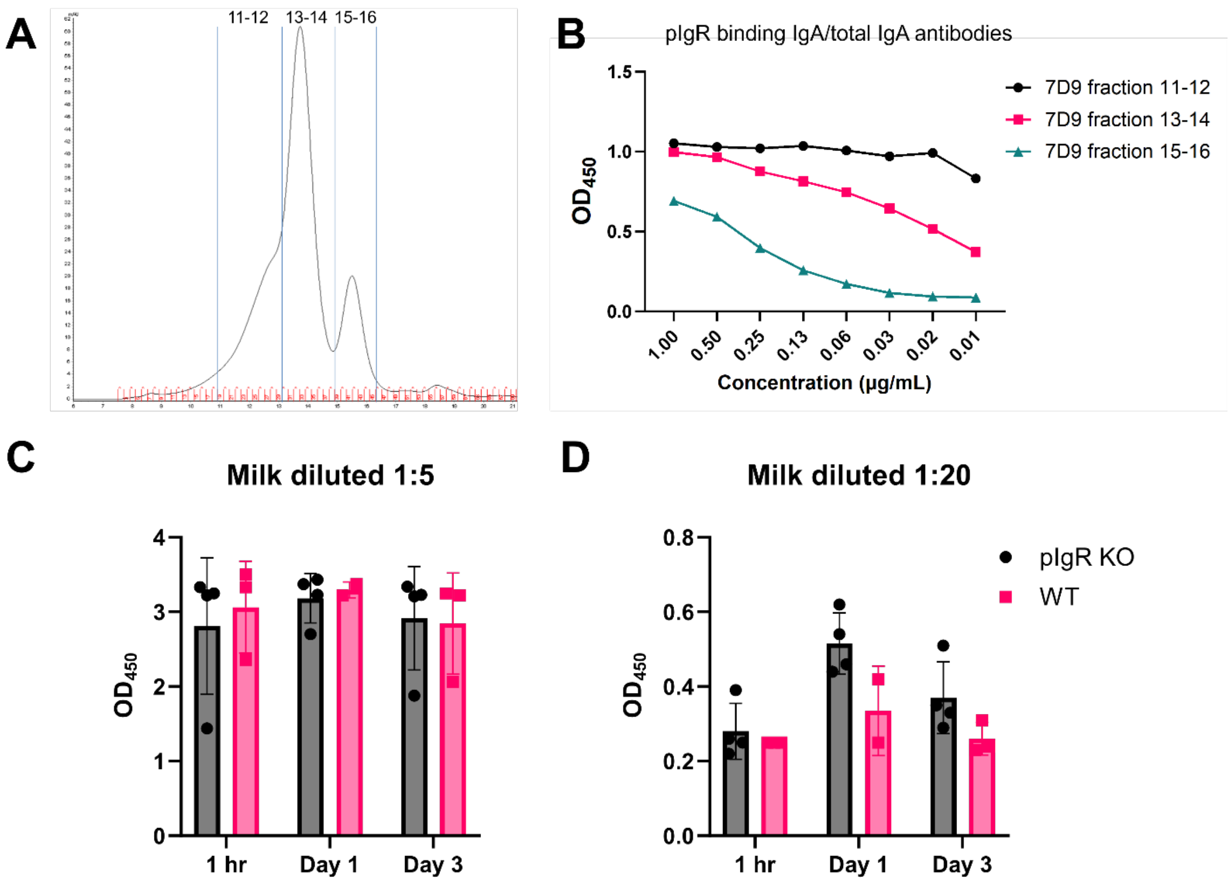


**Fig. S1. Characterization of hybridoma-produced 7D9 dIgA**. (A) Chromatogram of 7D9 using a Superose 6 10/300 GL on an AKTA liquid chromatography system. (B) Fractions collected from (A) were analyzed for polymeric immunoglobulin receptor (pIgR) binding IgA and total IgA antibodies using ELISAs across decreasing concentrations. The ratio of pIgR-binding IgA to total IgA is presented for each fraction (11-12 mL, 12-14 mL, 15-16 mL). The optical density (OD) values represent the mean of two technical replicates. (C) 5 mg/kg of dIgA 7D9 antibody was systemically injected into pIgR knockout (KO) or wild type (WT) C57BL/6 mice. VP6-specific IgA antibodies were measured in milk via a VP6 ELISA at 1:5 and (D) 1:20 dilutions. Each data point in (C) and (D) represents the optical density (OD) values of two technical replicates from one animal.

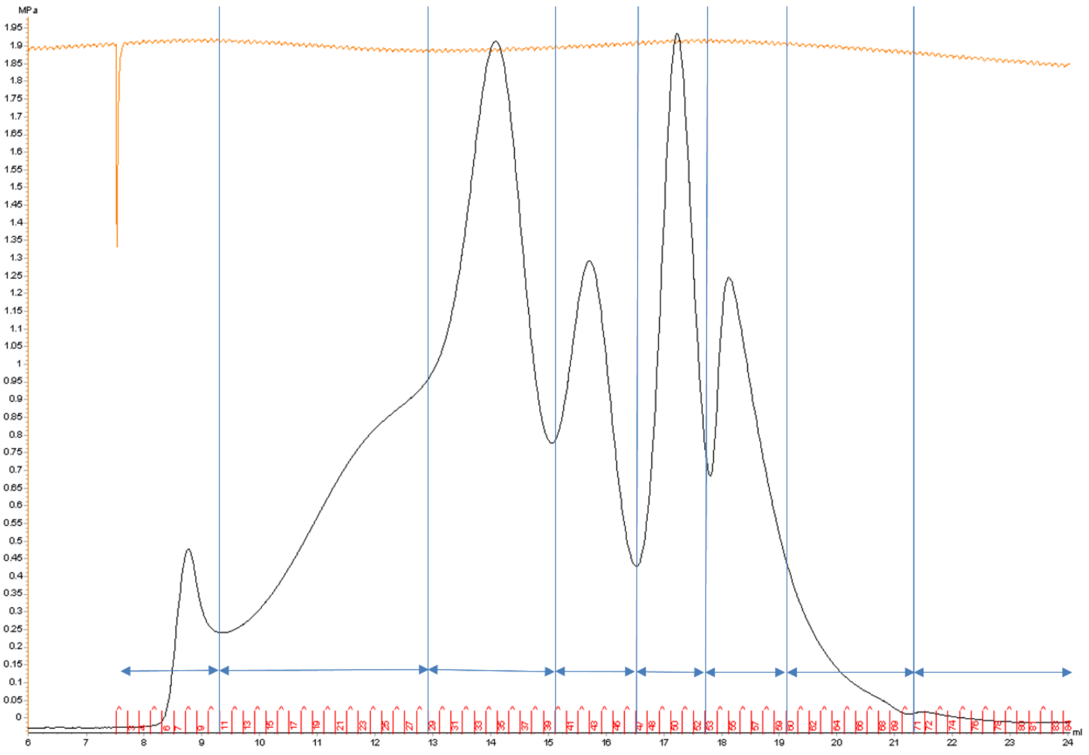

**Fig. S2. Size-exclusion chromatography revealed multiple species of IgA mAb41**. Chromatogram of mAb41 using a Superose 6 10/300 GL on an AKTA liquid chromatography system. The bottom x-axis (black numbers) shows the elution volume needed to elute IgA through the column, while the top x-axis (red numbers) provides additional subdivisions of the elution volume, enabling the collection of smaller, more precise fractions. The fractions tested in Figure 2D,E correspond to these finer, more accurate (red) fractions.

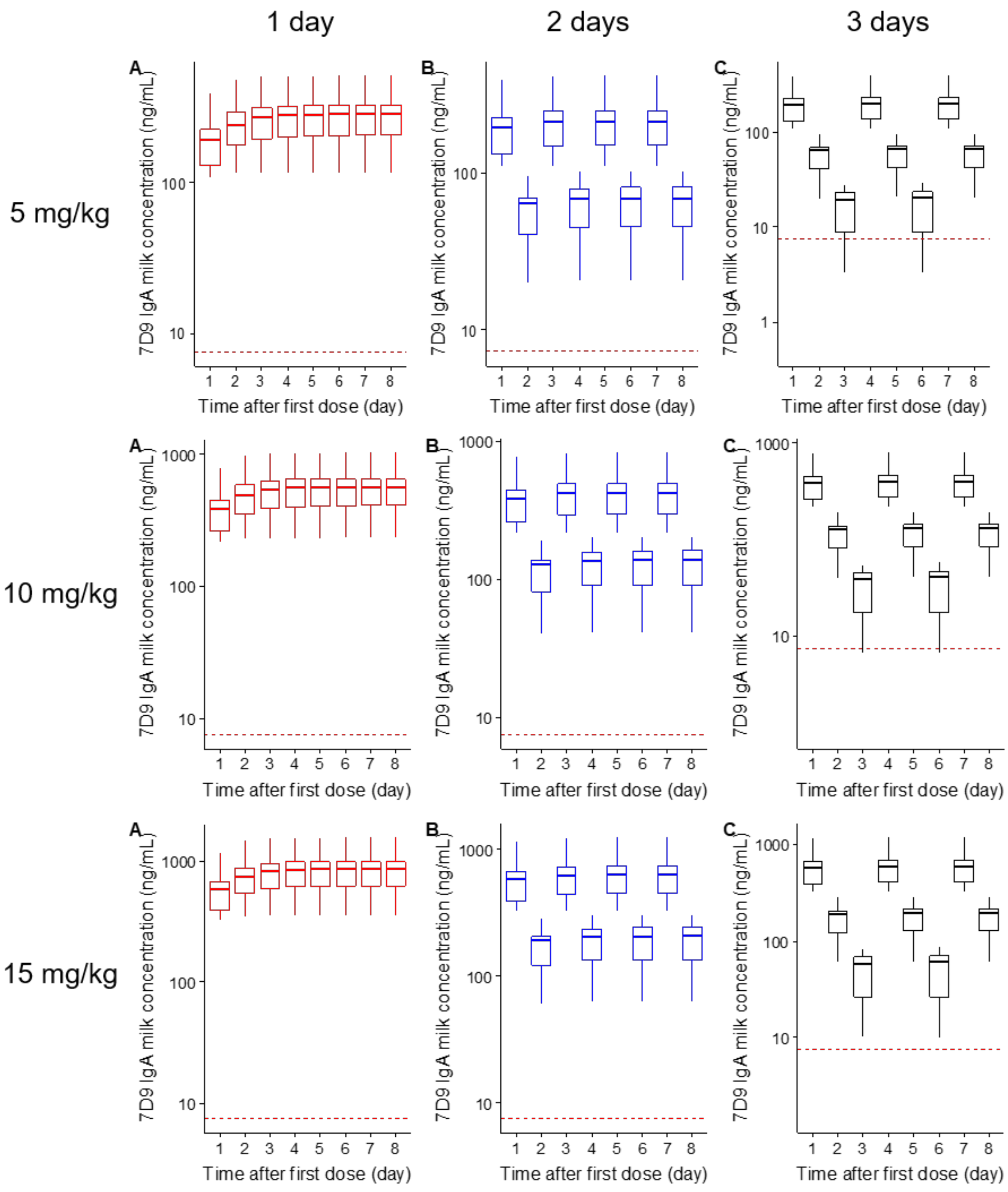


**Fig. S3. Pharmacokinetic (PK) analysis antibody levels in milk following intravenous 7D9 dIgA infusion.** (A) Empirical Bayesian estimates of individual PK parameters of blood and milk from 7D9 infused dams (main text Figure 1D) were used to simulate exposures following various doses of 7D9 IgA. Using a 1-day dosing interval, concentrations of 7D9 dIgA remained stable in milk for up to 8 days after the first dose (A). However, with the 2- (B) and 3-day (C) dosing intervals, 7D9 dIgA concentrations in

milk dropped by day 2 post-infusion and continued to decrease without an additional dose.

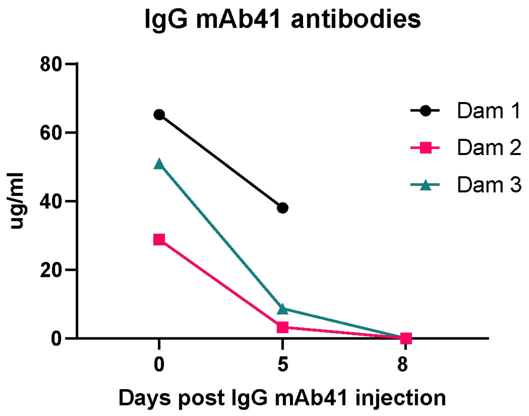


**Fig. S4. Concentrations of IgG mAb41 in milk after systemic infusion.** 129sv mice were tail vein injected with 5 mg/kg of IgG mAb41 and milk was collected at days 0, 5 and 8 post infusion. IgG mAb41 antibodies were measured by mAb41 anti-idiotypic antibody ELISA. Each data point represents the optical density (OD) values of two technical replicates from one animal.

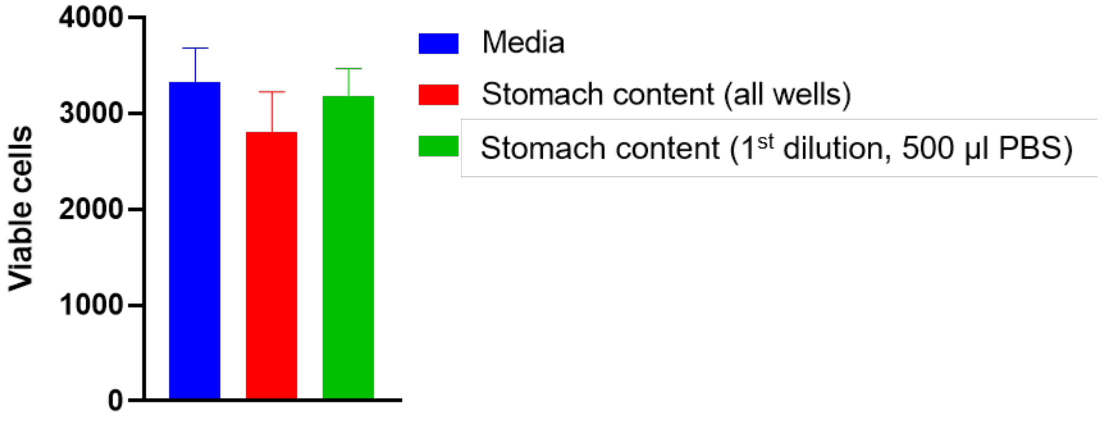


**Fig. S5. MA104 cell monolayer viability is not compromised by diluted stomach content samples.** There were no significant differences in MA104 cell viability between wells incubated with diluted (in 500 µl PBS) stomach content compared to medium in the RV infected cell binding assay. Data are plotted as the mean ± SD of two technical replicates.

**Figure 5**

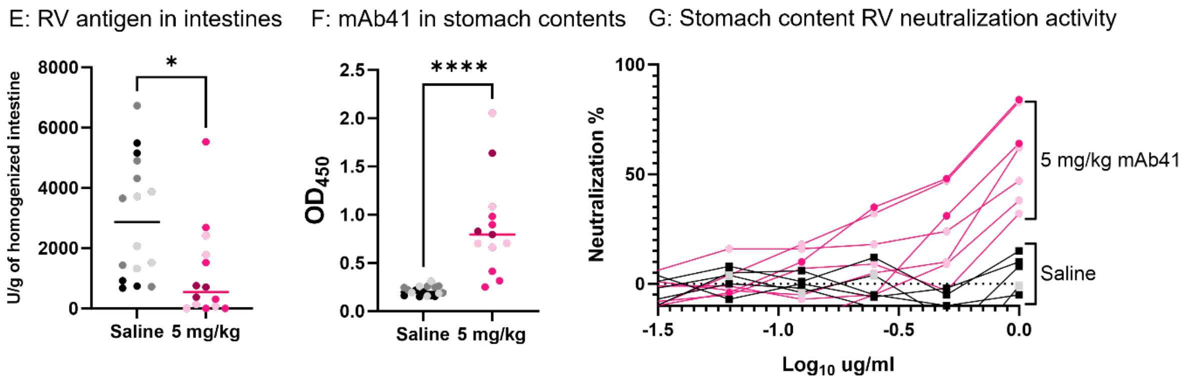


**Figure 6**

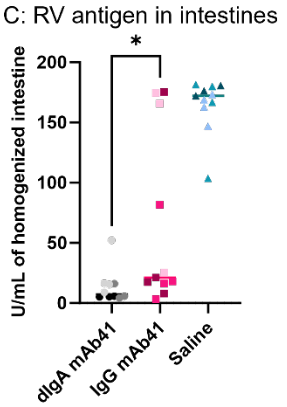


**Fig. S6. Litter distribution for assays in Figure 5 E-G and Figure 6C.**
